# Chronic Generalized Lymphadenopathy in a Child—Progressive Transformation of Germinal Centers (PTGC)

**DOI:** 10.3390/children9020214

**Published:** 2022-02-06

**Authors:** Carson Wills, Katherine Mercer, Jozef Malysz, Lidys Rivera Galvis, Chandrika Gowda

**Affiliations:** 1 Department of Graduate Education, Pennsylvania State University College of Medicine, Hershey, PA 17033, USA; cwills@pennstatehealth.psu.edu; 2 Department of Pediatrics, Pennsylvania State University College of Medicine, Hershey, PA 17033, USA; kmercer1@pennstatehealth.psu.edu; 3 Department of Pathology, Pennsylvania State University College of Medicine, Hershey, PA 17033, USA; jmalysz@pennstatehealth.psu.edu (J.M.); lrivera@pennstatehealth.psu.edu (L.R.G.)

**Keywords:** generalized lymphadenopathy, lymphoproliferative disease, colon polyp, Progressive Transformation of Germinal Centers

## Abstract

Background: Enlarged lymph nodes are a common complaint in a Pediatrician’s office. Diagnosis of reactive lymphadenopathy secondary to infectious, inflammatory, immune dysregulation calls for clinical investigation, including a thorough history, physical exam, imaging, and less often, a biopsy of the lymph node. Here we discuss a rare presentation of extensive generalized, chronic, waxing, and waning lymphadenopathy diagnosed as Progressive Transformation of Germinal Centers (PTGC) and the course of illness over eight years follow up period. Discussion: Progressive Transformation of Germinal Centers (PTGC) is considered a benign condition, but extensive recurrent generalized lymphadenopathy in a very young child has not been reported before. This case demonstrates the importance of long-term follow-up and tailoring the diagnostic work-up and management based on new signs and symptoms. Here we focus on the clinical considerations and management of complex presentation of a common clinical finding.

## 1. Introduction

Clinical presentation: A two-year-old caucasian male presented to the emergency department with a one-month history of a lump in the right axilla. No report of pain, redness, or open wound. However, the child has complained of discomfort, which prompted a visit to the Emergency Room. There was no history of fever, fatigue, weight loss, other swellings, or acute respiratory illness. No report of recent travel, contact with sick individuals, exposure to animals. The child’s father has a history of Large B-cell Lymphoma.

Physical exam revealed multiple enlarged lymph nodes in the anterior cervical, supraclavicular, bilateral axillary, and bilateral groin areas. The most prominent lymph node in the right axilla measured 2.5 cm in diameter. Complete blood count showed normal total white blood cell count, absolute lymphocyte count, neutrophil count, monocyte count, normal hemoglobin, and platelet count. Notably, the patient was positive for viral capsid antigen IgG and Epstein-Barr (EBV) nuclear antibody, indicating past EBV infection. A chest X-ray demonstrated no acute lung processes and no evidence of enlarged hilar lymph nodes. Ultrasonography of the right axilla showed numerous enlarged nodular structures compatible with abnormal lymph nodes comprised of a conglomerate mass. One of the larger superior nodes measured 2.6 × 1.5 × 1.8 cm. Multiple heterogeneous appearing lymph nodes demonstrated hypervascularity without definite fatty hila. This heterogeneous appearance was from multiple hypoechoic rounded areas within the parenchyma. Based on the clinical presentation, large size, and multiple locations of lymph nodes, the decision was made to biopsy the most prominent and accessible lymph node. An excisional right axillary lymph node biopsy was performed, which demonstrated reactive lymphoid hyperplasia with no evidence of lymphoma, bacteria, or fungal growth. The patient was advised to return for follow-up in six months.

**Figure 1:**
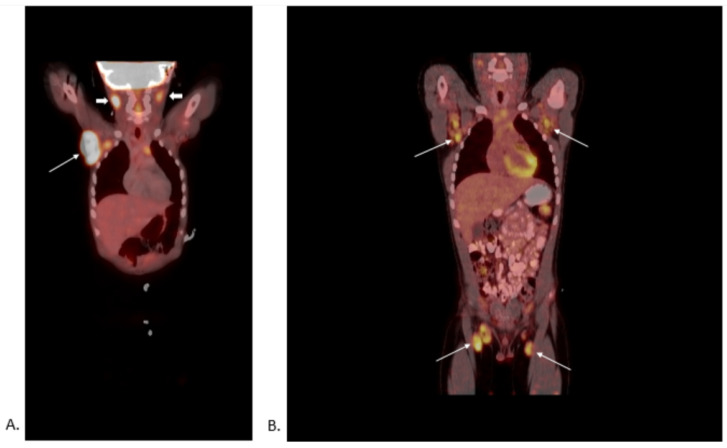
(**A**)**.** PET CT skull base to midthigh showing multifocal lymphadenopathy with the largest, most metabolically active node in the right axilla (arrow) Deauville score of 4. Other areas of increased uptake were noted in cervical lymph nodes (bold arrow), thymus, peri-aortic, retroperitoneal, and inguinal nodes, Deauville score of 4; 2 times the liver SUVmax. (**B**)**.** PET CT of skull base to midthigh showing persistent diffuse lymphadenopathy performed two years after initial presentation. Compared to an earlier study two years back, there was a stable to a mild decrease in metabolic activity in cervical, axillary (arrows) (arrows), iliac, and inguinal (arrows) lymphadenopathy, Deauville score of 4; 2 times the liver SUVmax. There was also stable to mildly increased left iliac lymphadenopathy. All lymph nodes were scored using Deauville criteria (5-point scale-Deauville Score [DS]) with a positive PET defined by tumor residual uptake moderately higher than liver (Deauville score of 4) or 2–3 times the liver SUVmax (Deauville score of 5). SUV-Standardized Uptake Value.

Upon follow-up after six months, he had persistent lymph nodes and newer palpable lymph nodes. The right upper extremity ultrasound showed multiple heterogeneous and hypervascular lymph nodes in the right axilla. Differential diagnoses considered at this point included lymphoma, granulomatous disease, or a benign lymphatic reactive process. He underwent a PET scan, which demonstrated FDG avidity in the pharyngeal tonsils and cervical lymph nodes, right axillary lymphadenopathy, several intensely FDG avid lymph nodes in the abdomen, and bilateral external iliac lymphadenopathy (Figure 1).

**Figure 2:**
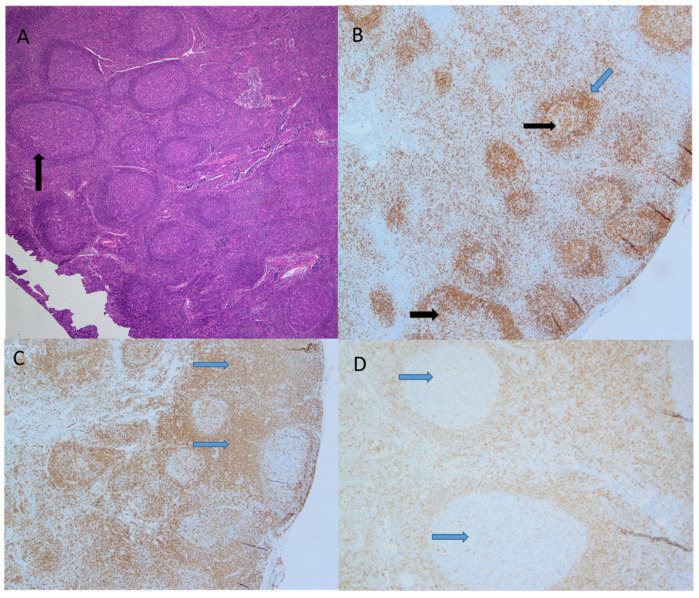
Pathologic evaluation of the lymph node demonstrated follicular and interfollicular hyperplasia with progressive transformation of germinal centers. (**A**). H&E staining showed intact nodal architecture with reactive follicles and occasional very large follicles showing progressive transformation of germinal centers; (**B**). PAX5 stain highlights B-lymphocytes within the reactive follicles with normal polarization and normal mantle zones, largest PTGC follicle shows encroaching of mantle zone lymphocytes on progressively transformed germinal center; (**C**). CD3 stain highlights T-cells and interfollicular hyperplasia; (**D**). BCL2 stain in negative in follicular center B-cells, consistent with benign reactive follicles.

Over the next four years, he presented with waxing and waning lymphadenopathy, which resulted in repeated imaging (PET/CT) and excisional biopsy on three separate occasions and two different health care facilities. Excisional lymph node biopsy showed follicular and interfollicular hyperplasia with Progressive Transformation of Germinal Centers (PTGC), with no evidence of lymphoma (Figure 2).

In this case, the primary differential considerations were Nodular Lymphocyte-Predominant Hodgkin lymphoma (NLPHL) and Pediatric type Follicular Lymphoma (PTFL). Preserved lymph node architecture and absence of distinct atypical cells of NLPHL, currently described as LP cells with characteristic folded or multilobulated nuclei and positivity for CD20, PAX5, and epithelial membrane antigen (EMA), did not support the diagnosis of NLPHL. Likewise, the absence of effacement of typical nodal architecture and staining pattern argued against the diagnosis of PTFL. In PTFL, lymph node architecture would be effaced or significantly distorted and replaced by expansile follicles, often with serpiginous growth patterns. Thus, the absence of morphologic and architectural features characteristic of PTFL did not support a malignant diagnosis. Flow cytometry showed that 5.9% of T-cells were positive for CD4 and CD8. T-cell gene rearrangement was negative. Bone marrow aspirate and biopsy demonstrated normocellular marrow with no clonal abnormality. The autoimmune proliferative syndrome (ALPS) diagnosis was considered, but Fas-mediated apoptosis testing was norma., CD4 and CD8 double-negative T-cell numbers were normal, ruling out ALPS. Evaluation by rheumatologist showed normal Anti-Neutrophil Cytoplasmic Antibody (ANCA), angiotensin converting enzyme (ACE), lysozyme, anti–Sjögren’s-syndrome-related antigen A (SSA) autoantibodies, also called anti-Ro and anti-SSB, rheumatoid factor, and total Creatine Phosphokinase (CPK) level were normal, suggesting against autoimmune or inflammatory disorder. Work-up for primary immunodeficiency disorders was also negative.

The IgG4-positive multiorgan lymphoproliferative syndrome is a distinct clinical entity characterized by elevated serum IgG level and increased IgG4 + plasma cell infiltration in involved tissues (pancreas, lacrimal and salivary glands, and lymph nodes) [1,2]. Studies have shown a difference in clinical and pathological findings between IgG4+ PTGC and IgG4− PTGC. Our patient did not have elevated serum IgG4 levels. Lymph nodes affected by IgG4+ PTGC show elevated IgG4+/IgG+ plasma cell ratio and increased involvement of submandibular glands [1]. IgG4 related disease is mainly described in older patients and reported in children with PTGC.

Cancers like acute leukemias, lymphoma, and even solid tumors like neuroblastoma can present with regional lymph node enlargement. A malignant lymph node is typically large, painless, firm, and immobile on an exam, primarily associated with constitutional symptoms (bruising, fever, malaise, and weight loss) and likely have abnormal blood indices (high or low white blood count, anemia, low platelets) [3]. The presence of any of these features in one or more lymph nodes should prompt referral to a specialist for further evaluation and consideration for biopsy [4,5]. Our patient did not have systemic symptoms except for mild fatigue.

Around age eight, he presented with recurrent and worsening abdominal pain. Colonoscopy on two separate occasions has revealed recurrent juvenile colon polyps measuring 20–24 mm removed. Genetic evaluation for Juvenile Polyposis Syndrome (JPS) and Li-Fraumeni Syndrome (*APC*, *BMPR1A*, *MUTYH*, *PTEN*, *SMAD4*, *STK11*, and *TP53* genes) were negative.

At present, the child continues to be thriving with age-appropriate growth and development. He continues to have multiple palpable pathologically enlarged lymph nodes. We continue periodic physical exams (every six months) to determine the need for further imaging such as ultrasonography and frequent discussion with family about overall clinical status, which has helped assess the need for imaging and or biopsy of the lymph node.

## 2. Discussion

Lymphadenopathy is a common physical exam finding in the pediatric population and is observed in approximately 55% of children under five who present for routine health maintenance visits [6,7,8]. The majority of pediatric cases are localized (52%) and self-limited (30%), and generalized lymphadenopathy is observed in about 18% of patients [9]. Lymphadenopathy is benign in most patients and is most frequently caused by a rash or systemic bacterial or viral infection [6,10,11].

Initial evaluation and diagnostic studies are based on the child’s age (younger age—more likely reactive), duration of symptoms (acute vs. chronic), and nature (size, number, location, signs of inflammation, pain) of the lymph node. History of associated symptoms (fever, upper respiratory symptoms) and exposures (travel, sick contacts, animals) will help decipher the etiology of lymphadenopathy. [3,12]. Laboratory evaluation is indicated when suspecting systemic cause and typically includes complete blood count, erythrocyte sedimentation rate (ESR).High platelet count and high ESR may indicate reactive thrombocytosis and systemic inflammatory reaction. Radiology exam including chest X-ray (cough, shortness of breath) to rule out mediastinal mass. Observation for 3–4 weeks is a reasonable approach in a young child with isolated lymph node enlargement without any signs and symptoms suggestive of severe systemic illness or malignancy [7,9]. A course of antibiotics in case of infectious etiology is helpful to document a decrease in size and response to treatment.

Corellating imaging findings with key clinical features is important for evaluating enlarged lymph nodes optimally. Ultrasonography (US) is a relatively cheap and noninvasive imaging modality to accurately determine the size, location, shape, clustering, homogeneity, and presence of fluid (abscess) within the lymph nodes [1]. This information, along with clinical information, can help in guiding the next steps, whether to proceed with watchful observation or biopsy. 18F-fluorodeoxyglucose (FDG) positron emission tomography-computed tomography (PET-CT) aid in the diagnosis or risk stratification of cancer-related lymphadenopathy [2,4]. However, its utility in differentiating malignant vs. non-malignant lymph nodes such as PTGC is limited [2]. PET/CT is a functional imaging modality using radiolabeled glucose probes which specifically bind and aggregate in target tissues with high metabolic activity. Malignant lymph nodes tend to have increased metabolic activity than normal surrounding tissues. However, non-malignant, reactive lymph nodes, including PTGC, can show high PET avidity, as seen in this patient [5]. Currently, there are no imaging modalities that can help differentiate PTGC from other reactive lymphadenopathies. The decision to proceed with biopsy of a persistant, large, PET-avid lymph node will need clinical co-relation and discussion with family.

Biopsy of lymph node, preferably excisional biopsy and histopathologic exam, is the gold standard to determine the diagnosis in case of extensive lymphadenopathy, especially when there is high clinical suspicion for malignancy [13,14]. The most common pathologic finding in biopsies from pediatric patients with lymphadenopathy is reactive follicular hyperplasia (52%), followed by granulomatous diseases (32%), neoplastic diseases (13%), and chronic lymphadenitis (3%) [6,7,15]. Reactive follicular hyperplasia is characterized by non-atypical enlargement of the germinal centers without disrupting typical lymph node architecture. The primary site of B-cell expansion in response to antigen stimulation is the germinal center of secondary lymphoid organs. Germinal centers are composed of a dark zone, where peripheral B-cells acquire antigen and undergo somatic hypermutation. A light zone contains a network of follicular dendritic cells that hold antigens and contribute to the germinal center selection of B-cells [16].

Progressive transformation of germinal centers (PTGC) is common in follicular hyperplasia, occurring in about 3.5–10% of patients with enlarged lymph nodes and reactive follicular hyperplasia [15,17,18,19]. PTGC is characterized histologically by large nodules in a background of follicular hyperplasia but usually accounts for only 5–10% of the affected follicle [16]. The PTGC lesions (within benign lesions) are isolated and scattered amongst reactive follicles (Figure 2). This likely explains the range of incidences in the literature as its diagnosis certainly depends on how extensive is the assessment of the lymph nodes (core vs. excisional biopsy) [20]. For example, if malignancy is suspected, more sections are likely submitted for histology and, thus, more likely to identify the scattered PTGCs [16].

Pathogenesis of the PTGC involves migration of mantle zone B-cells into the germinal center, causing germinal center fragmentation into centrocyte and centroblast islands and disruption of the follicular dendritic cell network [16,19]. Migration of mantle zone B-cells into the germinal center is preceded by T-cell invasion and redistribution, representing a normal stage in the resolution of follicular hyperplasia and follicular regression.

Clinically, PTGC demonstrates a 3:1 male predominance and is more common in children than adults in the United States [17,19,21]. PTGC is observed at an increased frequency in lymph node biopsies from patients with concurrent or prior NLPHL, and the risk of developing NLPHL is slightly increased in patients with PTGC [4]. Differentiating these diseases requires the histologic identification of lymphocytic and histiocytic tumor cells, which express CD20 and EMA [16]. No clear evidence is available regarding the risk of children with generalized lymphadenopathy with PTGC developing malignancy such as lymphoma in the future [3,19,20,21,22,23,24].

## 3. Conclusions

As demonstrated in this case and similar reports, PTGC presents with varied clinical presentation and poses diagnostic and management challenges. Prompt and thorough evaluation, biopsy of persistently enlarged lymph node followed by frequent monitoring, and a long-term follow-up plan are required. Furthermore, recurrent symptoms leading up to multiple lymph node biopsies demonstrate diagnostic, and management challenges that the clinicians have to face and family’s anxiety around the unknown risk of future malignancy. Although there are no clear guidelines for clinicians about the follow-up of lymphadenopathy due to PTGC, based on our patient experience, we learned that short interval follow-up and physical exam could guide further necessary work-up. In our case, a thorough physical exam every 4–6 months and discussion with the family about the child’s overall clinical status, growth, and development played a crucial role in formulating a reasonable follow-up plan to minimize imaging and invasive procedures while closely monitoring the disease course.

## Data Availability

Not applicable.
